# Paramedics, poetry, and film: health policy and systems research at the intersection of theory, art, and practice

**DOI:** 10.1186/s12960-019-0373-5

**Published:** 2019-08-07

**Authors:** Leanne Brady, Shaheem De Vries, Rushaana Gallow, Asha George, Lucy Gilson, Moira Louw, Abdul Waheem Martin, Khalid Shamis, Toni Stuart

**Affiliations:** 1 Health Policy and Systems Division, University of Cape Town, Cape Town, South Africa; 2 Emergency Medical Services, Health Department, Western Cape Provincial Government, Cape Town, South Africa; 3 School of Public Health, University of the Western Cape, Cape Town, South Africa; 4 Department of Global Health and Development, London School of Hygiene and Tropical Medicine, London, United Kingdom; 5 Tuba Films, Cape Town, South Africa; 6 Toni Stuart Poetry, Cape Town, South Africa

**Keywords:** Paramedics, Healthcare workers, Emergency medical services, Violence, Poetry, Film, Arts-based methods, Creative methods, Health policy and systems research, Ambulance

## Abstract

Violence is a public health issue. It is the consequence of a complex set of interacting political, social, and economic factors firmly rooted in past and current injustice. South Africa remains one of the most unequal countries in the world, and in some areas, the rates of violence are comparable to a country that is at war. Increasingly, paramedics working in high-risk areas of Cape Town are being caught in the crossfire, and in 2018, there was an attack on a paramedic crew nearly every week. These attacks are a symptom of much deeper, complex societal issues. Clearly, we require new approaches to better understand the complexity as we collectively find a way forward. It is in this context that we are collaborating with paramedics, poets, and filmmakers to tell human stories from the frontline thereby bringing the lived experiences of healthcare workers into policy making processes. In this commentary, we share a series of poems and a poetry-film that form part of a larger body of work focused on the safety of paramedics, to catalyze discussion about the possibilities that arts-based methods offer us as we seek to better understand and engage with complex social issues that have a direct impact on the health system.

**Electronic supplementary material:**

The online version of this article (10.1186/s12960-019-0373-5) contains supplementary material, which is available to authorized users.

## Main text

### Delivering emergency medical services (EMS) in a city of complex contradictions

Paramedics are negotiating unacceptable levels of violence as they deliver emergency medical care in high-risk areas of Cape Town, South Africa. In 2018, there were 64 reported violent attacks on ambulance crews [1] and with at least one incident every week, this is a health systems emergency. These incidents are a symptom of much deeper, complex societal issues but paramedics, the community health workers of emergency care, are literally getting caught in the crossfire. The impossible daily choices they face while delivering care put them at the heart of a “constitutional crisis” [2], they place their own lives at risk as they seek to uphold the right to emergency care. We are a country 25 years into an ostensibly peaceful democracy, but when paramedics leave home for a shift, they say goodbye to their families “as if [they are] going to war” [3].

Violence is, more generally, a significant public health issue. According to the World Health Organization’s world report on violence and health, 1.6 million people die from violence-related causes worldwide every year [4]. In South Africa, although health outcomes and mortality rates (for key areas such as maternal and child health) are improving across the country as a whole [5], violence remains endemic, and in the Western Cape province, specifically, the rates of violence are on the increase [6].

Yet violence is more than numbers on a graph, it is the consequence of a complex set of interacting factors firmly rooted in past and current injustice. In Cape Town, the legacy of apartheid continues to influence experiences of violence, with significant differences along race, class, and gender lines [7]. The links between violence and inequality are well described [8], and in South Africa, where inequality has actually got worse since the end of apartheid [9], high levels of violence should come as no surprise. With rates of violent death comparable to those in armed conflict zones [10], South Africa remains deeply unequal, and deeply divided, and these broader historical, social, and political factors have a significant influence on the health system.

It is in this complex and seemingly intractable setting that we are using poetry and film to help collectively re-imagine possibilities of finding a way forward. As part of a broader project of work focused on EMS safety, we have collaborated with paramedics, poets, and filmmakers to tell the human story of working on the road. While it is important to address the structural factors that perpetuate the injustices that are drivers for violence, all too often the lived experiences of people working at the frontline are just not part of the conversation. The series of poems and poetry-film were developed in conversation with paramedics—by joining them on shifts and riding around in ambulances over the period of a few months, and through a range of in-depth interviews to get a deeper understanding of their work, their personal stories, and the context in which they work.

### Lived experience (and storytelling) as important data for policy processes

Health systems can be very dehumanizing places, and if we are to take developing people-centered health systems [11] seriously, then the personal narratives of healthcare workers simply must be valued in our policy decision-making processes. The poetry and films are vivid reminders that in addition to the important work paramedics do, they are also people with families and lives. What clearly comes through is how their intrinsic motivation to provide a service to improve the situation in their communities forms a core part of their professional identity. That they do their work in contexts deeply affected by violence highlights both bravery, and the emotions of risk, vulnerability, and fear that are juxtaposed in the split second clinical decision-making required in emergency situations. Beyond the immediacy of violence and emergency responses, the lifelong repercussions of chronic stress and ill-health—incurred to ensure the well-being of others—cannot be ignored. The poetry and films not only bring issues of complexity and humanity of these paramedics to the fore, but also highlight the profound gratitude that underlies their safe return to their families at the end of a shift.

The experiences of violence are immediate, intimate, and gripping, but they are also deeply political, and prone to misinterpretation or narrow framing. Creative processes and visual media can offer valuable insights about this difficult and complex health phenomenon. These arts-based methods can, through a feast of the senses, create the possibility for deeper engagement that moves beyond health policy and research communities to reach a wider audience. However, it is important (1) to recognize that while these creative methods can be a powerful kick-starter to the conversation, this is only the beginning of a process of working collectively to develop new ideas and shared understandings and (2) to take a critical approach when creating visual and artistic representations of everyday realities to avoid perpetuating stereotypes that are unhelpful. While the experiences of violence are very real, the “danger of the single story” [12] is that we get stuck in narratives that are dis-enabling. This is where poetry and film can help shift the narrative so that we can start having a different discussion that puts the lived experiences of paramedics front and center of the conversation.

### Imagining possibilities through poetry and film: what are the implications for health policy and systems research?

So, how then might arts-based methodologies such as poetry and film offer new ways of influencing policy and research? Beyond recognizing that the lived experience and narratives of healthcare workers are legitimate forms of data, we also need to find ways to center them in policy making and research processes. Creative methods may help us do both.

Policy processes are influenced by multiple forms of evidence. However, the lived experiences of frontline health workers are often not considered as a legitimate input to policy decision-making, despite these experiences being central to how policies are implemented and experienced. Gathering this evidence—in ways that allow the experiences to be seen and heard clearly—is a fundamental part of the research needed to support the development of people-centered health systems [11].

In our work, arts-based methods have created platforms for discussion in a range of policy making processes. For example, a public screening of the film “Red Zone Paramedics” and a poetry performance by Toni Stuart brought together 200 residents (from a neighborhood with high levels of violence), paramedics, the director of EMS, a range of community activists and the local government ward counselors. The discussion that followed highlighted a number of important policy-relevant issues, but more importantly, allowed for a dialogue between senior managers, paramedics, and the people most affected by the policy decisions that are made. The arts-based methods were also used as part of a national EMS safety symposium where senior policy makers from across the country from multiple sectors (Provincial and National Department of Health, Labor, Health Professions Council) were brought together for a pre-symposium session entitled “Leadership in Conversation” organized by the Director of EMS. This session was held in a cinema and involved screening a series of films and then discussing realities on the ground and policy choices. At this event, copies of the films were requested for use in paramedic training. Subsequently, EMS created a position of an embedded researcher to support further participatory film making and reflection to strengthen organizational learning within the department. Across these events, the collaboration with poets and filmmakers has enabled forms of storytelling that allow a much deeper connection to the lived experiences of healthcare workers.

Creative methodologies such as poetry and film allow the lived experiences of health care workers to be centered in policy making processes. They give policy makers a window into the world of frontline providers and bring home the everyday realities of policy in practice. This could contribute towards new ways of thinking and new ways of doing in health systems. However, these possibilities will need to be tracked over time with research methodologies that capture the diverse experiences of the multiple stakeholders involved in co-production and viewing: community members, health workers and managers, artists, and researchers.

Finally, in our experience, arts-based methods have also been a vehicle to enhance the research process, supporting a research approach that puts participants in collaboration with researchers and policy makers. This has allowed the researcher to gain a much deeper understanding of the research context and will influence our methodological choices moving forward. Such an approach enhances the relevance of our work as engaged scholarship.

### Telling stories from the health system through poetry and film

In this section, we share poetry and a short documentary poetry-film done as part of the broader body of work. “After the Night” a film directed by Leanne Brady and edited by Khalid Shamis is available as Additional file 1 and a series of poems about paramedic life by Toni Stuart can be found below (full English translation has been shared as Additional file 2).


**Additional file 1:** “After the Night” film directed by Leanne Brady and edited by Khalid Shamis and is available here: https://vimeo.com/304310322. (MP4 1 112 424 kb)

Poetry Paramedics July - August 2018 | CAPE TOWN by Toni Stuart

**about the night**

die nag het oë and you can’t see it

hulle hou vir jou dop uit ie donkerte uit

die valley se beacon is rooi vanaand

en ie tafel se gesig, sig weer

want haar kinders verdwyn

in ie son se afgaans

behind a closed door, a daughter

buttons up her green shirt

pulls on her green trousers

and laces up her black boots

her parents remind her to pray

and that they will pray too. simple words

whispered against the stillness of night

to keep their daughter safe.

outside another front door, a mother

tells her children: “when i leave here

it could be the last time you see me.

when i come back, it’s a privilege.”

die nag het oë and you can’t see it

hulle hou vir jou dop uit ie donkerte uit

the van’s bright lights stare back

into the dark that has eyes, searching for safety

no lights will flash red here. no sirens

will wake another mother’s sleep

want die valley se beacon is rooi vanaand.

staal is ‘n ding is koud. the daughter sees a man

place the cold weapon on the front seat of her van.

in a different van, the mother argues

with her partner who is driving,

that Eastridge is the colour of blood

“let’s go to the police station”

and wait for an escort,” she says.

but waiting is 20 mins, is 30 mins, 40 mins

her partner says no Eastridge is the colour of sunset

and he drives into that orange flare

en die nag se oë hou om dop

daai kinders wat verdwyn het

met ie son se afgans, kryp weg hier onder

Eastridge se fading light and they return now

their arms have grown steel branches

en hulle wag, hulle wag vir die van

met ie groen en geel strepe

met ie rooi letters wat hardop praat

en hulle time, hulle time ie van se stop

“it’s a load and go,” says the daughter.

“we’re not treating them at home.”

“we treat while we’re driving.”

want die nag het oë and you can’t see it

hulle hou vir jou dop uit ie donkerte uit.

**a heart in a green uniform**

1.

I was a machinist in a factory for 21 years,

she tells me. there is no rain this morning

–the sun is a simple yellow promise rising

cold over the Plain’s streets and homes.

I volunteered for 7 long years. In that 7

years, I was married, I had 2 kids,

I had a full-time job, but I was always available for a shift.

it’s been 20 years since she first climbed

into an ambulance. 20 years in service,

and she has long since traded the safety

and routine of the shop floor for the thrust

of chest compressions and 12-hour road

trips. Now, she is a grandmother of three,

and treating children is the hardest part of the job.

If a child gets hurt or ill-treated or mistreated,

I forget what I am: I become a mother.

If a kid gets knocked down you want to bring them back.

outside, the winter is remembering how

to bring back Cape Town’s rain. it falls

over the Plain, the same way it falls over

the rest of the city.

ek hou van my werk, man.

ek hou van my werk. I always say

you can take what out of my scope

you can take away my vehicle

you can take away my uniform

but here, she points to her heart,

here, I am a paramedic

2.

the daughter, whose parents remind her to pray

before she steps out on night shift,

was 19 the first time she climbed into a van.

19, on a ride along and the first call

from the control room said gunshot wound.

he was 19, and I was 19, she says

he died on the scene, even though the paramedics

did everything they could.

that first call, up until today, it stayed with me.

the daughter, who is also a sister

is treating a patient at their house, the first time

she is attacked.

I got to the vehicle and there was a guy scratching

through our things, and

he put a gun on the seat. she throws her cellphone

into the cubby hole, turns around

to see four men in front of her, their faces covered

their hands holding knives.

the men search her and her partner, take everything

that was the first thing I was thinking about: please, don’t rape me.

it is years later but each time she drives into Tafelsig,

the palpitations return. she laughs

I laugh but that is just how I cope with things.

she bakes too, cakes and other

things, she bakes more now than before, it helps

more than the tablets do.

the daughter who is also sister, who is also an aunt

hopes to be a mother someday.

3.

in Tafelsig, a father leaves his baby daughter at home

to go to work. he steps out of his front door in green.

he remembers a call from years ago: the control room

sent them to Kanonkop, where a mother gives birth

to a baby not breathing. he and his partner arrive

at the house: the baby is on her mother’s stomach,

but there is no pulse. they wrap the baby. his partner

sees to the mother. he takes the baby to the front

of the ambulance, puts the heaters on full blast and

starts resuscitations. he calls the paramedic on shift,

don’t wait on the scene, he says, start driving and meet me

halfway. ek is ver van jou af, maar ek is op pad.

my partner sit haar voet neer en ons gly. we make

our way to the hospital. when we came to the MOU, that child was crying.

you know how relieved we felt that day it’s a phenomenal

feeling. years later, a woman walks into the husband

who is yet to be a father, in the Town Centre, she asks

him if he knows her. he says no. the woman points

at the girl standing next to her, Remember that baby in Kanonkop, this is her.

the girl is 4.

That was rewarding, the husband who is now a father says.

To see that she made it, she grew up and she’s gorreloos, you know.

**after the night**

her vehicle pulls into the base and stops

she climbs out and walks to the kitchen

she asks who wants coffee and sets out the cups.

the dark still has its hold on the sky

she makes her own cup of coffee last.

when it’s done, she greets everyone

and climbs into her own car.

she drives out of the base.

at the exit of Lentegeur Hospital

she turns left and drives straight towards

the east, not five minutes later and

she is home. she climbs out of her car

and sits on the green grass in front

of her house. she watches the sun rise

it’s a sign of gratitude she says.

just sitting with a cup of coffee,

my full compos mentis, my house is

still standing here, my daughter opens

the door. my little ones are gone to school

it’s a lot really, a lot to be grateful for

the sun rises over the Plain differently

than it does over the rest of the city

the first rays of yellow light are softer

here, as if this Mother City knows

that her children here are grown from a different love.

## Conclusions

In conclusion, arts-based methods are increasingly used in socially engaged research practice to better understand, and engage with complex social issues [13]. We too have found that poetry and film are a powerful way to open up a collaborative space in which to have a conversation about the safety of paramedics. We have seen this in engagements ranging from poetry performances and film screenings followed by public discussions, to film nights included in high-level policy discussions as part of national symposia. Health systems are complex adaptive systems—they are non-linear open systems, and changes in one part of the system can have unexpected ripple effects across the system [14]. They are characterized by emergence (where changes are not determined by individual system components, but rather through interactions between components) and the pathways of change triggered are unpredictable. Consequently, the policy and system effects of these moments of collective reflection and sensemaking [15] are as yet unknown. However, poetry and film have opened up possibilities for new ways of thinking and doing, and as we are already seeing changes on the ground, we look forward to tracking where the conversation leads from here in terms of policy and system changes. Stakeholders concerned with the safety of paramedics must hear multiple views about these experiences and so there is value in creating spaces that allow for sharing stories, imagining possibilities and new ways of thinking to find a way forward.

## Additional files

Additional file 2: Full English poetry translation. (DOCX 26 kb)

## Abbreviation

EMS: Emergency medical services
