# Supplementary material for: Paramedics, poetry, and film: health policy and systems research at the intersection of theory, art, and practice
Source: Hum Resour Health. 2019 Aug 7;17:64. doi: 10.1186/s12960-019-0373-5 (PMC6686373; doi:10.1186/s12960-019-0373-5)
Supplement: Supplementary file 2 — Full English poetry translation. (DOCX 26 kb) [file 12960_2019_373_MOESM2_ESM.docx]

**POETRY PARAMEDICS**

July - August 2018 | CAPE TOWN

by Toni Stuart

| **about the night** |
| --- |
| the night has eyes, and you can’t see it |
| it watches you from deep within its darkness |
|  |
| Beacon’s Valley is red tonight |
| and Tafelsig’s face sighs once again |
| as her children disappear |
| with the setting sun |
|  |
| behind a closed door, a daughter |
| buttons up her green shirt |
| pulls on her green trousers |
| and laces up her black boots |
|  |
| her parents remind her to pray |
| and that they will pray too. simple words |
| whispered against the stillness of night |
| to keep their daughter safe. |
|  |
| outside another front door, a mother |
| tells her children: “when i leave here |
| it could be the last time you see me. |
| when i come back, it’s a privilege.” |
|  |
| the night has eyes, and you can’t see it |
| It watches you from deep within its darkness |
|  |
| the van’s bright lights stare back |
| into the dark that has eyes, searching for safety |
| no lights will flash red here. no sirens |
| will wake another mother’s sleep |
|  |
| because the Beacon’s Valley is red tonight. |
| steel is a cold hard thing. |
| place the cold weapon on the front seat of her van. |
| in a different van, the mother argues |
| with her partner who is driving, |
| that Eastridge is the colour of blood |
| “let’s go to the police station |
| and wait for an escort,” she says. |
|  |
| but waiting is 20 mins, is 30 mins, 40 mins |
| her partner says no Eastridge is the colour of sunset |
| and he drives into that orange flare |
| and the eyes in the night follow his tracks |
|  |
| those children who disappeared |
| with the sun’s descent, are hiding here in |
| Eastridge’s fading light |
| their arms have grown steel branches |
|  |
| waiting for the van |
| with the green and yellow stripes |
| with the loud red letters |
| and they’re timing it’s stop |
|  |
| “it’s a load and go,” says the daughter. |
| “we’re not treating them at home. |
| we treat while we’re driving.” |
|  |
| the night has eyes, and you can’t see it |
| It watches you from deep within its darkness |
|  |

**a heart in a green uniform**

1.

| I was a machinist in a factory for 21 years, |  |
| --- | --- |
| she tells me. there is no rain this morning |  |
|  |  |
| –the sun is a simple yellow promise rising |  |
| cold over the Plain’s streets and homes. |  |
|  |  |
| I volunteered for 7 long years. In that 7 |  |
| years, I was married, I had 2 kids, |  |
|  |  |
| I had a full-time job, but I was always available for a shift. |  |
| it’s been 20 years since she first climbed |  |
|  |  |
| into an ambulance. 20 years in service, |  |
| and she has long since traded the safety |  |
|  |  |
| and routine of the shop floor for the thrust |  |
| of chest compressions and 12-hour road |  |
|  |  |
| trips. Now, she is a grandmother of three, |  |
| and treating children is the hardest part of the job. |  |
|  |  |
| If a child gets hurt or ill-treated or mistreated, |  |
| I forget what I am: I become a mother. |  |
|  |  |
| If a kid gets knocked down you want to bring them back. |  |
|  |  |
| outside, the winter is remembering how |  |
| to bring back Cape Town’s rain. it falls |  |
|  |  |
| over the Plain, the same way it falls over |  |
| the rest of the city. |  |
|  |  |
| i love my work, man. |  |
| i love my work. I always say |  |
|  |  |
| you can take what out of my scope |  |
| you can take away my vehicle |  |
| you can take away my uniform |  |
| but here, she points to her heart, |  |
|  |  |
| here, i am a paramedic |  |

2.

the daughter, whose parents remind her to pray

before she steps out on night shift,

was 19 the first time she climbed into a van.

19, on a ride along and the first call

from the control room said gunshot wound.

he was 19, and I was 19, she says

he died on the scene, even though the paramedics

did everything they could.

that first call, up until today, it stayed with me.

the daughter, who is also a sister

is treating a patient at their house, the first time

she is attacked.

I got to the vehicle and there was a guy scratching

through our things, and

he put a gun on the seat. she throws her cellphone

into the cubby hole, turns around

to see four men in front of her, their faces covered

their hands holding knives.

the men search her and her partner, take everything

that was the first thing I was thinking about: please, don’t rape me.

it is years later but each time she drives into Tafelsig,

the palpitations return. she laughs

I laugh but that is just how i cope with things.

she bakes too, cakes and other

things, she bakes more now than before, it helps

more than the tablets do.

the daughter who is also sister, who is also an aunt

hopes to be a mother someday.

3.

| in Tafelsig, a father leaves his baby daughter at home |
| --- |
| to go to work. he steps out of his front door in green. |
|  |
| he remembers a call from years ago: the control room |
| sent them to Kanonkop, where a mother gives birth |
|  |
| to a baby not breathing. he and his partner arrive |
| at the house: the baby is on her mother’s stomach, |
|  |
| but there is no pulse. they wrap the baby. his partner |
| sees to the mother. he takes the baby to the front |
|  |
| of the ambulance, puts the heaters on full blast and |
| starts resuscitations. he calls the paramedic on shift, |
|  |
| halfway. I’m far away from you, but I’m on my way |
|  |
| my partner puts her foot down and we glide. we make |
|  |
| you know how relieved we felt that day it’s a phenomenal |
| feeling. years later, a woman walks into the husband |
|  |
| who is yet to be a father, in the Town Centre, she asks |
| him if he knows her. he says no. the woman points |
|  |
| the girl is 4. |
|  |
| That was rewarding, the husband who is now a father says. |
| To see that she made it, she grew up and she’s mischievous, you know. |
|  |
|  |

**after the night**

her vehicle pulls into the base and stops

she climbs out and walks to the kitchen

she asks who wants coffee and sets out the cups.

the dark still has its hold on the sky

she makes her own cup of coffee last.

when it’s done, she greets everyone

and climbs into her own car.

she drives out of the base.

at the exit of Lentegeur Hospital

she turns left and drives straight towards

the east, not five minutes later and

she is home. she climbs out of her car

and sits on the green grass in front

of her house. she watches the sun rise

it’s a sign of gratitude she says.

just sitting with a cup of coffee,

my full compos mentis, my house is

still standing here, my daughter opens

the door. my little ones are gone to school

it’s a lot really, a lot to be grateful for

the sun rises over the Plain differently

than it does over the rest of the city

the first rays of yellow light are softer

here, as if this Mother City knows

that her children here are grown from a different love.

*AUTHOR’S NOTE*

*These poems were commissioned by Leanne Brady, a medical doctor and public health systems*

*activist & researcher, doing a cross-institutional PHD at the University of the Western Cape and*

*the University of Cape Town in Health Policy and Systems Research.*

*Funding was received from the South African Research Chair in Health Systems, Complexity and*

*Social Change supported by the South African Research Chair's Initiative of the Department of*

*Science and Technology and National Research Foundation of South Africa (Grant No 82769). Any*

*opinion, finding and conclusion or recommendation expressed in this material is that of the writer*

*and the NRF does not accept any liability in this regard.*

*To write the poems, I spent time interviewing three paramedics who live and work in*

*Mitchell’s Plain, Cape Town. The interviews were around their experiences of working in Red*

*Zones - areas where paramedics have been attacked and thus now require a police escort before*

*they can respond to a call in that area. Two of the paramedics interviewed live in Red Zones*

*themselves. Many of the words used, in addition to any direct quotes, are words that the*

*paramedics used themselves while sharing their stories with me.*
